# Dynamic cellular remodeling and symbiont genetic heterogeneity during early bacterial colonization in the deep-sea mussel *Bathymodiolus septemdierum*

**DOI:** 10.1186/s40851-026-00271-w

**Published:** 2026-08-21

**Authors:** Ayu Yuasa, Tetsuro Ikuta, Yoshihiro Takaki, Miwako Tsuda, Yuki Morono

**Affiliations:** 1 Institute for Extra-Cutting-Edge Science and Technology Avant-Garde Research (X-Star), Japan Agency for Marine-Earth Science and Technology (JAMSTEC), 2-15 Natsushima, Yokosuka, Kanagawa 237-0061 Japan; 2 Graduate School of Marine Science and Technology, Tokyo University of Marine Science and Technology, 4-5-7 Konan, Minato-ku, Tokyo, 108-8477 Japan; 3 Project Team for Development of New-Generation Research Protocol for Submarine Resources, Japan Agency for Marine-Earth Science and Technology (JAMSTEC), 2-15 Natsushima, Yokosuka, Kanagawa 237-0061 Japan; 4 Kochi Institute for Core Sample Research, Japan Agency for Marine-Earth Science and Technology (JAMSTEC), 200 Monobe-Otsu, Nankoku, Kochi 783-8502 Japan

**Keywords:** Chemosynthetic symbiosis, Deep-sea mussels, *Bathymodiolus septemdierum*, Symbiont colonization, Cellular remodeling, Genetic heterogeneity, Array tomography, Single-cell amplicon sequencing, Laser microdissection, In situ hybridization

## Abstract

Symbiotic relationships between animals and microorganisms are widespread and have driven the adaptation and evolution of animals across diverse environments. The establishment of highly integrated endosymbioses often involves dynamic cellular remodeling and management of symbiont populations. Deep-sea mussels of the subfamily Bathymodiolinae horizontally acquire chemosynthetic bacteria in specialized gill cells called bacteriocytes, primarily at the posterior ends of their gills, where new filaments are continuously formed throughout their lifetime. This group exhibits a wide diversity of symbiotic modes, ranging from extracellular to strictly intracellular, and hosts diverse symbionts, such as methane- and sulfur-oxidizers. To identify the universal mechanisms underlying stable symbiosis from this diversity, it is crucial to investigate how these partnerships are initiated. However, the cellular-level processes underlying the initial symbiont colonization and integration remain elusive in all species of this group. To elucidate this early phase, we employed three-dimensional volume electron microscopy, single-cell amplicon sequencing, and in situ hybridization targeting a specific symbiont genomic locus to examine the initial symbiont colonization process in the deep-sea vent mussel *Bathymodiolus septemdierum*, focusing on host cell morphology and symbiont population structure. Three-dimensional ultrastructural reconstruction revealed that initial colonization follows a specific and characteristic structural remodeling process distinct from typical phagocytosis, demonstrating how this early cellular reorganization leads to the complex architecture of mature bacteriocytes. Complementing this morphological observation, two independent but mutually supportive approaches-single-cell amplicon sequencing and in situ hybridization-collectively indicated that these early colonizing cells harbor a genetically diverse mixture of symbiont strains. These findings challenge the previously proposed single-cell uptake hypothesis for this species and suggest that host cells initially acquire diverse symbiont subpopulations from the environment. We propose that the clonal symbiont populations observed in mature individual cells may result from an intracellular purification process potentially linked to host cellular remodeling during colonization. The integration of 3D ultrastructural observations and single-cell molecular data provides a foundation for deciphering the universal mechanisms of stable symbiosis, paving the way for a deeper understanding of animal–microbe interactions at the subcellular level.

**Supplementary Information:**

The online version contains supplementary material available at https://doi.org/10.1186/s40851-026-00271-w.

## Background

Symbiotic associations between animals and microorganisms are ubiquitous and have profoundly shaped animal evolution, adaptation, and diversity [1]. These associations exhibit a broad spectrum of spatial localization, ranging from extracellular relationships to intracellular symbioses, in which microbes reside within specialized host cells, such as bacteriocytes [1]. Establishing these highly integrated symbiotic states requires large-scale cellular reorganization and dynamic morphological remodeling of host cells during the initial colonization of microbes [2, 3]. This acquisition is not a passive entry but an active host-driven process involving cytoskeletal rearrangements and extreme plasma membrane deformations, which is generally considered to occur via the co-option of phagocytosis in intracellular associations [2, 4]. For instance, in extracellular symbioses, colonization of the squid *Euprymna scolopes* by *Vibrio fischeri* induces a four-fold increase in microvillar density, supported by abundant F-actin to envelop the bacteria [5]. In intracellular associations of cnidarians, such as corals and anemones, host phagocytic cells actively engulf endosymbiotic dinoflagellates into specialized intracellular vesicles [6].

In horizontally transmitted symbioses, where hosts acquire their symbionts from the environment every generation, hosts are often colonized by diverse environmental bacterial strains. Acquiring a diverse array of symbionts provides the host with access to greater symbiont genetic diversity and allows for flexible local adaptation to fluctuating environments, as seen in deep-sea hydrothermal vent snails and mussels [7, 8]. However, accommodating a mixed symbiont population often requires the host to manage complex intracellular population dynamics. Indeed, dynamic shifts in symbiont composition, driven by within-host selection and population bottlenecks, have been observed in various animals, such as squids, insects, and deep-sea bivalves [9–11]. Consequently, elucidating the dynamic morphological changes of host cells during the onset of microbial colonization is of critical scientific importance to understand how this early cellular remodeling leads to the formation of specialized mature bacteriocytes. Furthermore, it provides a critical foundation for exploring how this physical cellular remodeling might relate to the initial structuring of symbiont populations during the establishment of stable mutualism.

Deep-sea mussels of the subfamily Bathymodiolinae serve as excellent models for studying this intricate host-microbe interplay. These mussels horizontally acquire chemosynthetic symbionts into their gills throughout their lifetime [12]; however, their symbiotic modes exhibit a wide diversity across species, ranging from extracellular to fully intracellular associations [13–15]. Recent studies of specific lineages have provided valuable insights into these symbioses. For instance, genomic studies on *Gigantidas platifrons* have revealed the adaptive expansion of host immune and endocytotic gene families to accommodate symbionts [16]. Furthermore, cell biological and transcriptomic analyses of *G. japonicus* have demonstrated the roles of phagocytosis and mTORC1 in symbiont digestion and maintenance [17–19], while 3D volume electron microscopy of *G. platifrons* has revealed complex, interconnected channel systems within mature bacteriocytes [20]. Although these findings highlight the sophistication of mussel-microbe interactions, the diversity in symbiotic modes among bathymodioline mussels indicates that the mechanisms observed in one species may not necessarily apply to others. In *Bathymodiolus septemdierum*, previous structural and molecular analyses have revealed unique cellular characteristics at the host-microbe interface. Mature bacteriocytes feature an intricate, interconnected vesicular structure that maintains open pathways to the external environment [21] (Fig. S1). In this open-ended physical architecture, the symbiont population inhabiting each bacteriocyte is highly restricted and mostly clonal [21], whereas diverse strains co-occur within a single host individual to form a mosaic localization pattern in the gills [8].

These observations raise a fundamental question: how are this intricate, open-ended cellular architecture and the highly restricted clonal population coordinately established during the initial stages of symbiont colonization? To explain the formation of this unique cellular state, a hypothetical model was previously proposed for *B. septemdierum*: a nascent host cell initially takes up only a “single” bacterial cell via a phagocytic process, and as this founding symbiont proliferates, the enveloping host membrane branches and expands to accommodate the growing population while maintaining its connection to the exterior [21]. However, directly testing this “single-cell uptake and membrane branching” hypothesis has remained technically challenging. Conventional two-dimensional (2D) electron microscopy struggles to capture the full three-dimensional (3D) complexity of large-scale membrane deformations during the early stages of colonization. Furthermore, bulk-tissue genetic analyses and conventional fluorescence in situ hybridization (FISH) lack the spatial and genomic resolution required to track the strain-level composition and sorting dynamics of symbionts within individual nascent bacteriocytes. Consequently, the exact mechanisms orchestrating host cell development and the initial structuring of the symbiont population remain unclear.

To overcome these technical limitations and empirically elucidate the sequence of events during symbiosis establishment, we employed an integrated multimodal approach to investigate nascent bacteriocytes in *B. septemdierum*. First, we utilized three-dimensional volume electron microscopy (array tomography) to reconstruct the large-scale morphological remodeling of the host cell membrane during initial symbiont colonization. To complement these ultrastructural findings and resolve the genetic composition of these newly acquired symbiont populations in situ without bulk tissue bias, we developed a single-cell genomics approach combining laser microdissection (LMD) with amplicon sequence variant (ASV) analysis, coupled with direct-geneFISH [22, 23] targeting a specific symbiont genomic locus. A previous study on closely related *Bathymodiolus* species has shown that new gill filaments are continuously generated at the posteriormost budding zone (bz) throughout the host’s lifetime, and as the gills grow, these newly formed filaments are sequentially displaced anteriorly. The study has also demonstrated that initial symbiont colonization reproducibly begins at a specific developmental stage of these tissues, typically around the eighth filament anterior to the bz, making symbiont colonization a continuous process throughout the individual’s lifespan [12]. Consequently, this spatial arrangement represents a chronological sequence of host cell development. By exploiting this arrangement, we traced the progression of early-stage symbiont colonization. Through this combination of 3D ultrastructural and single-cell genomic analyses, we tested the previously proposed single-cell uptake hypothesis. Based on these results, we explore the potential relationship between host cell morphodynamics and the initial structuring of the symbiont population.

## Methods

### Animal sampling

*B. septemdierum* was collected from hydrothermal vent sites on Myojin Knoll, Japan, during cruises KS-20–1 (January 7–11, 2020) with the remotely operated vehicle (ROV) *Hyper Dolphin* operated by the research vessel (R/V) *Shinsei-Maru* of the Japan Agency of Marine-Earth Science and Technology (JAMSTEC) at depths of 1182 and 1232 m, KM22-15 (December 3–20, 2022) with the ROV *KM-ROV* operated by the R/V *Kaimei* of JAMSTEC at a depth of 1222 and 1224 m, and YK23-12 (July 11–18, 2023) with the human-occupied vehicle (HOV) *Shinkai 6500* operated by the R/V *Yokosuka* of JAMSTEC at a depth of 1237 m.

For whole-mount FISH, single-cell sequencing, and direct-geneFISH, after cutting the adductor muscle, juveniles with a shell length of 5–7 mm were fixed in 4% paraformaldehyde in 1× phosphate-buffered saline (PBS) at 4 °C, followed by stepwise dehydration in an ethanol series and then stored at − 30 °C. For electron microscopy, the juveniles were fixed in 2.5% glutaraldehyde in seawater filtered with a 0.2-μm filter unit (Nalgene, Rochester, NY, USA) (filtered seawater, FSW) at 4 °C. Additionally, the gills of adult mussels were excised using a disposable scalpel, and were fixed in 4% paraformaldehyde as described above for direct-geneFISH.

### Whole-mount fluorescence in situ hybridization

Whole-mount FISH was performed as previously described [24] with some modifications. Four juveniles were sagittally bisected, and the soft bodies were excised from the shells. Hybridization was performed in 100 µL of the hybridization mixture containing 20 mM Tris -HCl, 0.9 M NaCl, 5 mM EDTA, 0.01% SDS, 20% formamide, and 0.5 µM of the probe designed for symbiont 16S rRNA of *B. septemdierum* (Bsob692; 5’-CGCCATTGATGTTCCTTCAG-3’ [25]) labeled with Alexa Fluor 488 at 46 °C for 2 h. Following hybridization, juveniles were washed twice in a solution containing 20 mM Tris-HCl, 225 mM NaCl, 5 mM EDTA, and 0.01% SDS for 20 min at 48 °C and counterstained with 2 µg/mL 4′,6-diamidino-2-phenylindole (DAPI) in 1 × PBS for 3 min at room temperature (RT). After counterstaining, the juveniles were mounted in Vectashield Antifade Mounting Medium (Vector Laboratories, Newark, CA, USA). Sagittal Z-stack images covering the entire thickness of the posterior region of the gill were acquired using a Leica Stellaris STED system (Leica Microsystems, Wetzlar, Germany) in confocal mode. Image acquisition and subsequent 3D rendering were performed using the system’s control software, Leica Application Suite X (LAS X) v. 4.8.2 (Leica Microsystems).

### Transmission electron microscopy

For transmission electron microscopy (TEM), the excised gills of an adult mussel were fixed in 2.5% glutaraldehyde in FSW at 4 °C. Subsequent sample preparation, resin embedding, ultrathin sectioning, and TEM observation were performed as previously described [26].

### Tomographic three-dimensional reconstruction and surface observation of gill cells

Scanning electron microscopy (SEM) and subsequent 3D reconstruction of the gill cell images were performed as previously described [21]. Briefly, the juvenile soft body, with its shells removed and sagittally bisected, was embedded in Quetol resin. To ensure sectioning stability, the resin block was trimmed to a small face targeting the gill area around the eighth filament anterior to the bz, and approximately 300 sagittal serial ultrathin sections with a thickness of 80 nm were prepared from one juvenile individual. After staining with uranyl acetate and subsequent osmium coating, the sections were observed under an FEI Quanta 450FEG electron microscope (Thermo Fisher Scientific, Waltham, MA, USA) operated at 7 kV. Five cells containing a small number of symbiotic bacteria were selected around the eighth filament anterior to the bz at the posterior end of the gill. To cover the entire cell thickness, 78–132 serial sections were photographed at × 20000 magnification for each of the five cells. Materials of interest (symbionts, cell nuclei, and cell membranes) were manually traced for segmentation throughout the image stack using the image analysis software Microscopy Image Browser v. 2.84 (MIB [27]), and 3D tomographic images were constructed using AMIRA v. 6.3 (Thermo Fisher Scientific) with post-processing, including surface rendering and smoothing. Additionally, host cell volumes were calculated using the surface area volume function of AMIRA. The correlation coefficient between the number of symbiotic bacteria and host cell volume was calculated as a descriptive statistic using Microsoft Excel (v16.82; Microsoft, Redmond, WA, USA).

To observe the apical surface of the gill cells using SEM, the gill filaments around the eighth filament anterior to the bz were individually separated from four juvenile individuals, prepared for SEM as previously described [21], and observed under an FEI Quanta 450FEG electron microscope (Thermo Fisher Scientific) operated at 5 kV.

### Single-cell preparation using laser microdissection (LMD)

After sagittal bisection and shell removal, the soft bodies of the four juvenile individuals were pre-embedded in 1% Agarose, Type VII: Low Gelling Temperature (Sigma-Aldrich, St. Louis, MO, USA) to preserve their morphology. The samples were then embedded in Paraplast (Leica Biosystems, Richmond, IL, USA) or cryopreserved in O.C.T. compound (Sakura Finetek Japan Co., Ltd., Tokyo, Japan). To cover the entire cell thickness, 15-μm-thick sagittal sections were prepared using a microtome onto 2.0-µm or 4.0-µm PEN membranes (Leica Microsystems) with an aluminum frame. After removing the paraffin or O.C.T. compound, the sections were stained with SYBR Green I Nucleic Acid Gel Stain (Lonza, Rockland, ME, USA) diluted 1:1000 in 1 × PBS for 3 min at RT, washed three times with 1 × PBS for 2 min each at RT, and embedded in 20% n-propyl gallate in 10% glycerol in PBS. Sections were observed at × 93 magnification, and the entire cell thickness was acquired as Z-stacks using a Leica Stellaris STED system (Leica Microsystems). We specifically selected target gill cells located around the eighth filament anterior to the bz that contained a small cluster of symbiont signals (approximately 3–30 symbiont cells). To ensure the complete recovery of all symbionts within a single target cell and to prevent the accidental inclusion of symbionts from neighboring cells during subsequent laser microdissection, we carefully confirmed that these target signals were spatially isolated with sufficient signal-free margins in all three dimensions (x, y, and z axes). To ensure that these nucleic acid signals exclusively represented the symbionts, we preliminarily confirmed by FISH using a symbiont-specific 16S rRNA probe [25, 26] labeled with Alexa Fluor 488 and a general bacterial probe (EUB338 [28]) labeled with Alexa Fluor 594, combined with DAPI staining of juvenile sections of the posterior region of the gill including the bz. This demonstrated that all DAPI-positive signals in the bacteriocytes (excluding the host cell nuclei) completely colocalized with both FISH signals, indicating the absence of non-symbiotic bacteria (Fig. S2). The gills were also imaged using a low-magnification lens, and the locations of the target cells were recorded. Following observation, the sections were washed three times with 1 × PBS at RT for 15 min each and coated with 0.1% gelatin to prevent tissue shrinkage during the subsequent drying process required for LMD, which could otherwise distort the localization of the fluorescent signals.

Using the images acquired during the initial confocal microscopy as a visual guide, each target cell was individually identified using a laser microdissection system, LMD6500 (Leica Microsystems) equipped with a fluorescence mode, and approximately 10 × 10 µm regions of seven target single cells encompassing the entire symbiont signals in the cell were then excised into separate PCR tubes. As a reference, three gill areas (approximately 100 × 100 µm) located more anteriorly and containing multiple host cells that harbor numerous symbiotic bacteria were excised and collected in separate PCR tubes. Furthermore, four areas outside the tissue between the gill filaments, approximately 100 × 100 µm in size, where no SYBR green signals were observed, were also excised and collected in separate tubes. All samples were stored at − 80 °C until use.

### Amplicon sequencing analysis of symbiont genes within single bacteriocytes

To serve as the template for the first round of PCR, the microdissected samples collected into PCR tubes were lysed in a 1.25-µL reaction mixture containing 20 mM Tris-HCl (pH 8.0), 5 mM EDTA, 400 mM NaCl, 0.3% SDS, and 200 µg/mL of Proteinase K (Takara Bio, Shiga, Japan) for 3 h at 55 °C, followed by 95 °C for 5 min. Multiplex PCR was performed using primer sets for the symbiont specific *ribE* and *proB* genes as markers for sequence polymorphism as previously described [21]. The reaction was performed in a 50-µL mixture containing 25 µL of Ampdirect Plus (Shimadzu, Kyoto, Japan), 1.25 µL of the lysis reaction mixture, 1.25 U of BIOTAQ HS DNA polymerase (Meridian Bioscience, Cincinnati, OH, USA), 0.2 µM of the primer sets for *ribE* and *proB*, and UltraPure Distilled Water (Thermo Fisher Scientific, Waltham, MA, USA). The first-round reaction consisted of 10 min at 95 °C and 35 cycles of 94 °C for 30 s, 54 °C for 30 s, and 72 °C for 20 s. Amplification of the two genes was confirmed by electrophoresis, and the amplified products were purified twice using Agencourt AMPure XP beads (Beckman Coulter, Brea, CA, USA) and ExoSAP-IT PCR Product Cleanup Reagent (Thermo Fisher Scientific) at 37 °C for 30 min and 85 °C for 15 min, and were subjected to a second round of PCR. A second round of PCR was performed to add multiplexing indices and Illumina and AVITI sequencing adapters using Ex Taq polymerase (Takara Bio). The reaction consisted of 1 min at 96 °C, 15 cycles of 96 °C for 30 s, 65 °C for 45 s, and 72 °C for 1 min, followed by incubation at 72 °C for 7 min. The adapter-attached PCR products were purified twice using Agencourt AMPure XP beads and quantified using the Qubit 1X dsDNA HS Assay Kit (Thermo Fisher Scientific, Waltham, MA, USA). Paired-end sequencing (2 × 300 bp) was performed using an AVITI system (Element Biosciences, San Diego, CA, USA). Amplicon sequence data were processed as previously described [29], and then amplicon sequence variants (ASVs) were generated using the DADA2 plugin [30] wrapped in QIIME2 v. 2025.7.0 pipeline [31]. For all samples, ASVs whose maximum relative abundance exceeded 10% were selected for further analysis. The alignments were performed using the MAFFT [32] v. 7.52 and NJ tree were constructed using BioNJ [33] implemented in SeaView v. 5.1 [34]. The raw reads were deposited in the DDBJ Sequenced Read Archive under accession numbers DRR1076222–DRR1076235 in BioProject PRJDB42758.

### Direct-geneFISH

Direct-geneFISH was performed as previously described [23, 35] with the following modifications: PCR was performed with Phusion High-Fidelity DNA Polymerase (Thermo Fisher Scientific) using a specific primer set designed for the membrane-bound hydrogenase (*hup*) gene cluster as a marker for the presence or absence of this genomic locus. This cluster has been previously confirmed to exhibit heterogeneity in its presence or absence, depending on the subpopulation of the *B. septemdierum* symbiont [8]. The 50-µL reaction mixture contained 1 × Phusion Buffer, 2 mM dNTPs, 0.5 µM of the primer set (Table S1) with 15-base adaptor sequences required for subsequent In-Fusion cloning, 1.0 U of Phusion High-Fidelity DNA Polymerase, and 1 ng of genomic DNA of the *B. septemdierum* symbiont prepared in a previous study [8] as the template. The reaction consisted of 30 s at 98 °C, followed by 30 cycles of 98 °C for 10 s, 58 °C for 30 s, and 72 °C for 2 min 30 s, followed by an extension at 72 °C for 10 min. The 4675-bp amplicon was cloned into the pBluescript II KS (+) vector (Agilent Technologies, Santa Clara, CA, USA) using an In-Fusion HD Cloning Kit (Takara Bio). Ten genomic regions of 300–324 bp in the *hup* gene cluster, corresponding to those used as templates for direct-geneFISH in a closely related mussel species (*B. azoricus*) in a previous study [35] were selected as templates for direct-geneFISH in this study and amplified using PCR. The reaction mixture (100 µL) contained 1 × Phusion Buffer, 2 mM dNTPs, 0.5 µM of the ten primer sets designed for the ten template regions (Table S1), and 2.0 U of Phusion High-Fidelity DNA Polymerase, with purified plasmid DNA containing the *hup* gene cluster serving as a template. The reaction consisted of 30 s at 98 °C, followed by 30 cycles of 98 °C for 10 s, 58 °C for 30 s, and 72 °C for 2 min 30 s, with a final extension at 72 °C for 10 min. Amplification was confirmed by electrophoresis, and the products were purified using the Wizard SV Gel and PCR Clean-Up System (Promega, Madison, WI, USA). The probes were labeled with Alexa Fluor 594 using Ulysis Nucleic Acid Labeling Kit (Thermo Fisher Scientific), as previously described [23]. An artificial sequence probe (NonPolyPr350 [35]), labeled with Alexa Fluor 594 as described above, was used as a negative control.

For hybridization, after sagittal bisection and removal of the shells, a piece of the gill from one adult mussel and soft bodies of four juveniles were embedded in paraffin and three 4-µm sagittal sections per individual were prepared. After dewaxing, permeabilization was performed in 100 µL of permeabilization mixture containing 0.5 mg/mL lysozyme, 1 × PBS, 0.05 M EDTA, and 0.1 M Tris-HCl (pH 8.0) at 37 °C for 30 min. The sections were photobleached in 1 × PBS for 2 h using an autofluorescence quenching illuminator (TiYO; Nepa Gene, Chiba, Japan) and treated with TrueBlack Lipofuscin Autofluorescence Quencher (Biotium, Fremont, CA, USA) in 70% formamide for 30 s, followed by three washes in 1 × PBS at RT for 2 min each. For probe denaturation, the hybridization mixture prepared as previously described [23, 35] was incubated at 85 °C for 15 min and then immediately placed on ice. For denaturation of genomic DNA in the tissue, the sections were incubated in 70% formamide in 0.1% SSC at 50 °C, 70% formamide in diethyl pyrocarbonate (DEPC)-treated water at 4 °C, and 35% formamide at 4 °C for 3 min each. Subsequently, 50 µL of the hybridization mixture was applied onto the sections, and hybridization was performed in a humidified chamber at 46 °C overnight. After hybridization, the sections were washed in a solution containing 0.07 M NaCl, 0.02 M Tris-HCl (pH 8.0), 5 mM EDTA, and 0.01% SDS for 5 min and then for 15 min at 48 °C, and then transferred to 1 × PBS for 20 min at RT. The sections were counterstained with 5 µg/mL DAPI in 1 × PBS for 3 min at RT and embedded in ProLong Glass (Thermo Fisher Scientific). The sections were observed using a Leica Stellaris STED system (Leica Microsystems) in confocal mode and the micrographs were processed using Adobe Photoshop 2026 v. 27.8.0 (Adobe Inc., San Jose, CA, USA).

## Results

### Cell morphology during the early stages of symbiont colonization

**Fig. 1:**
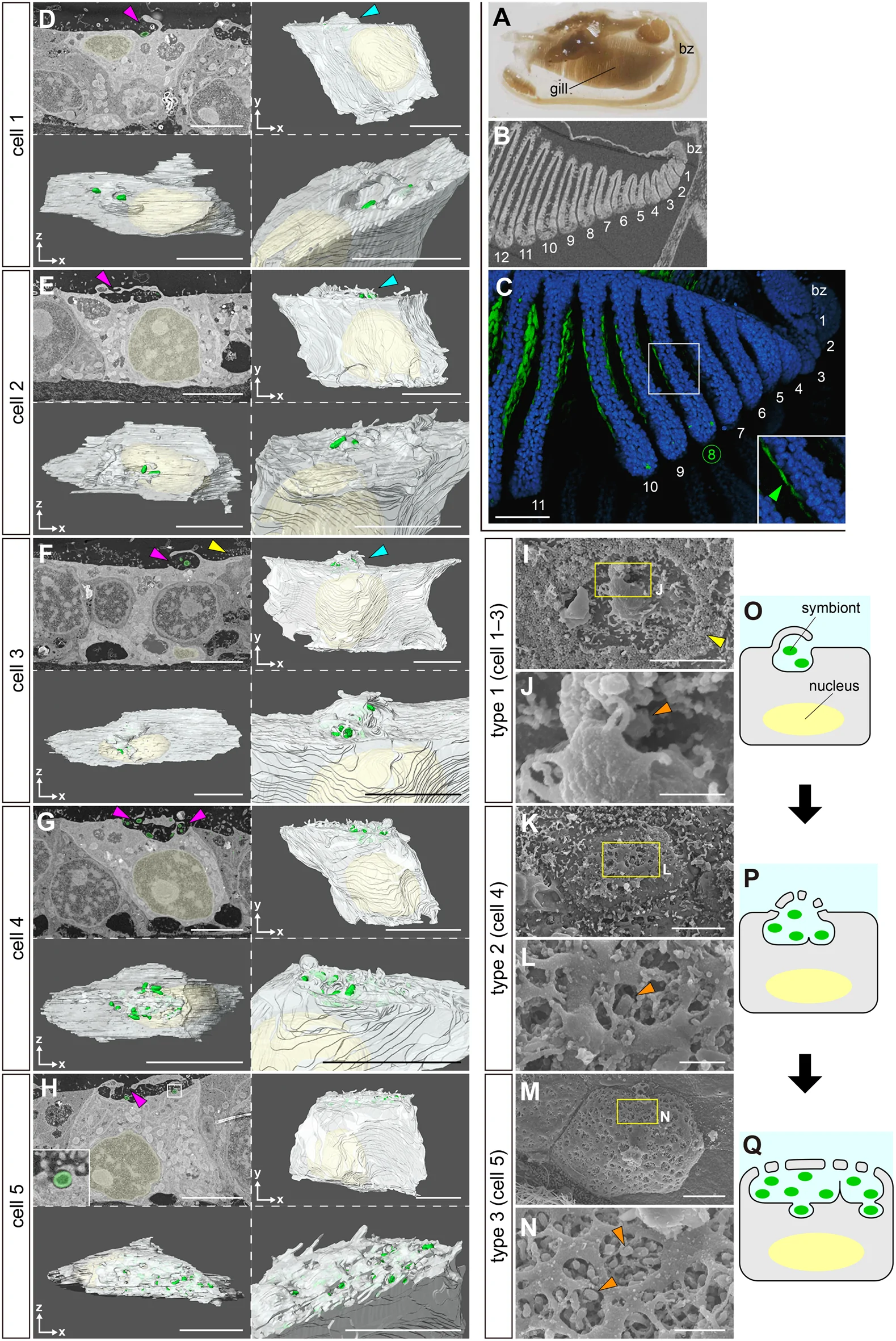
Cellular remodeling of *Bathymodiolus septemdierum* during the early symbiont colonization. (**A**) Sagittally bisected soft body of juvenile *B. septemdierum*. (**B**) SEM image of a posterior juvenile gill section. (**C**) Whole-mount fluorescence in situ hybridization (FISH) image of the posterior juvenile gill targeting symbiont 16S rRNA (green) with DAPI (blue) (3D projection of confocal z-stacks); Scale bar: 100 µm. Inset shows a higher magnification image. Posterior side is at the right in (**A**–**C**). (**D**–**H**) Three-dimensional (3D) reconstructions of host cells (cells 1–5), showing cell morphologies during early symbiont colonization. Each panel consists of four sub-panels: (top-left) a representative ultrathin section used for the 3D reconstruction (apical side up); (bottom-left) an apical view of the 3D model; (top-right) a lateral view of the 3D model (apical side up); and (bottom-right) a slightly magnified 3D view from an optimal angle to illustrate the morphological features during colonization. White arrows indicate the x, y, and z axes. Symbionts are labeled in green; cytoplasm and nucleus are labeled in white and yellow, respectively, and are made slightly transparent. Magenta, light blue, and yellow arrowheads indicate apical openings, apical protuberances, and microvilli, respectively. Inset in (**H**) shows a higher-magnification image of a small chamber formed by constriction. Scale bars: 5 µm. (**I**–**N**) Representative SEM images showing the three distinct types of apical surface morphologies corresponding to the stages observed in the 3D reconstructions. Panels J, L, and N are higher-magnification views of the regions indicated by the yellow rectangles in panels I, K, and M, respectively. Yellow arrowheads indicate microvilli on the surrounding cells. Light blue, magenta, and orange arrowheads indicate protuberances, openings, and bacteria-like structures, respectively. Scale bars: 1 µm (**J, L**), 5 µm (**I, K**), 10 µm (**M**), and 2 µm (**N**). (**O**–**Q**) Schematic cross-sectional diagrams illustrating the three stages of host cell remodeling during early symbiont colonization

To validate the spatial and temporal progression of symbiont colonization in *B. septemdierum*, we first performed whole-mount FISH targeting bacterial 16S rRNA in the gills of four juvenile individuals. Consistent with previous observations in closely related *Bathymodiolus* species [12], Z-stack 3D imaging of the posterior part of the gill revealed a reproducible spatial pattern across all examined individuals: an uncolonized bz, followed by the initiation of symbiont signals around the eighth gill filament anterior to the posteriormost bz, which then extended anteriorly into fully colonized filaments (Fig. 1A–C).

**Fig. 2:**
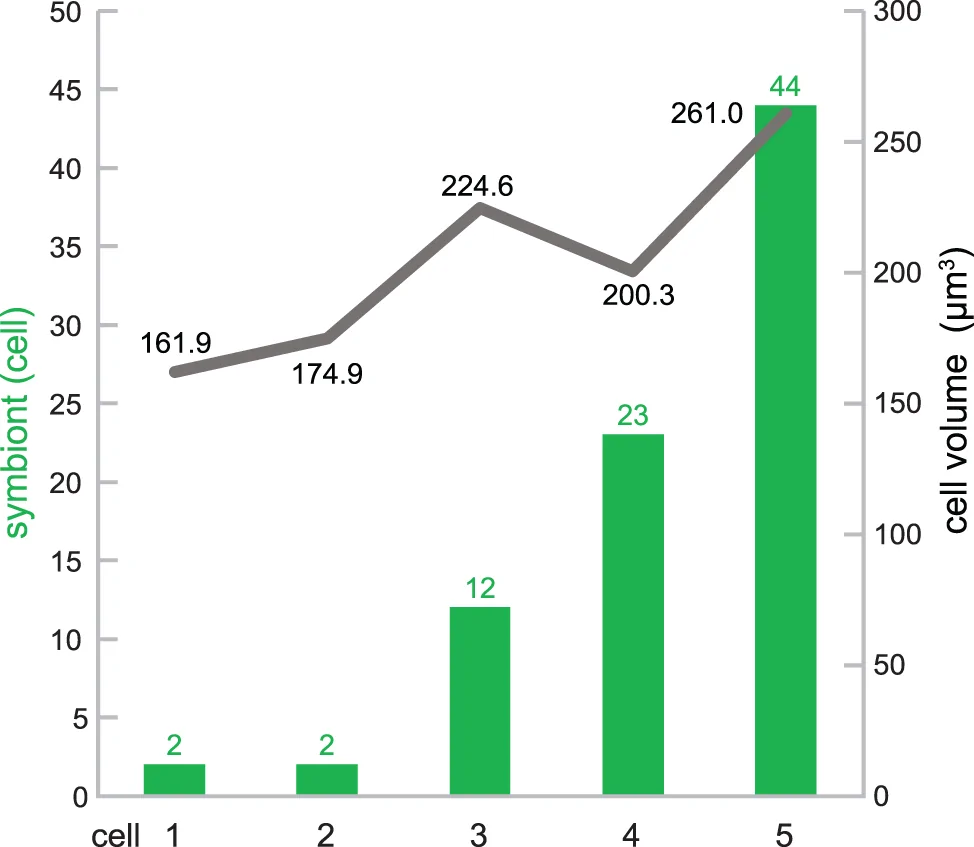
Relationship between symbiont count and host cell volume during the early stages of colonization. The horizontal axis represents the representative host cells (Cells 1–5) corresponding to the 3D reconstructions shown in Fig. 1. The left vertical axis and green bars indicate the number of symbiotic bacteria observed within the interconnected cavity of each cell. The right vertical axis and gray line indicate the corresponding volume of the host cell (µm^3^). The specific values for symbiont count and cell volume are annotated above each data point. A descriptive positive association was observed between the symbiont count and cell volume across these five representative cells (*r* = 0.88)

To reveal the cell morphology during the colonization of symbiotic bacteria in *B. septemdierum*, five host cells (cells 1–5) containing a small number of symbiotic bacteria were selected around the eighth gill filament anterior to the posteriormost bz (Fig. 1B) of a single juvenile individual, and 3D reconstructions were performed (Fig. 1D–H). Cell 1 (Fig. 1D; Movie S1) exhibited pseudopod-like structures extending from its apical surface, forming an isolated and localized protuberance. Beneath this structure, the plasma membrane was invaginated to form a cavity that remained continuous with the extracellular environment through a few openings. Two symbiont cells were observed in the cavity. Cell 2 exhibited a morphology similar to that of cell 1 (Fig. 1E; Movie S2), containing the same number of symbiotic bacteria (two), but with a slightly larger volume (Fig. 2). Similarly, cell 3 (Fig. 1F, Movie S3) had a distinct protuberance on its apical surface with a cavity beneath it, which was also connected to the outside through some openings. Cell 3 had a larger volume than cells 1 and 2, and 12 symbionts were observed within the cavity (Fig. 2). Additionally, some host cells adjacent to cells 1–3 had numerous microvilli on their apical surfaces (Fig. 1F). Cell 4 (Fig. 1G; Movie S4) exhibited an apical protuberance similar to those of cells 1–3, but it was lower and broader, occupying approximately half of the apical surface of the cell. This broadened structure possessed many openings that were directly connected to the cavity beneath it. Although the 2D cross-sections appeared to show multiple isolated cavities beneath the surface, the 3D reconstruction demonstrated that they actually constituted a single interconnected cavity undergoing compartmentalization. Twenty-three symbiont cells were observed in this cavity. Cell 5 (Fig. 1H; Movie S5) had a larger volume than cell 4, and its entire apical surface exhibited a slightly swollen appearance with numerous openings. Beneath this surface, the compartmentalization of the cavity progressed further, and multiple narrower chambers, each enclosing only a single symbiont, were formed by constriction. 3D reconstruction demonstrated that the main cavity, its compartments, and these smaller chambers constituted a single interconnected space, which maintained continuous contact with the external environment through the apical openings. Forty-four symbiont cells were observed within this complex interconnected structure. Overall, these five host cells contained between 2 and 44 symbiont cells in these interconnected spaces, with host cell volumes ranging from 161.9 to 261.0 µm^3^ (Fig. 2). A descriptive positive correlation was observed between the number of symbiotic bacteria in the cavity and host cell volume across the five examined cells (*r* = 0.88). These structural and anatomical features suggest that the morphological changes in host cells during the early stages of symbiont colonization can be classified into three distinct stages (cells 1–3, cell 4, and cell 5; Fig. 1O–Q). Regarding the spatial relationships among the reconstructed cells, cells 1, 2, and 5 were located within the same gill filament (around the eighth filament anterior to the posteriormost bz), whereas cell 3 was derived from the adjacent posterior filament (one filament posterior) and cell 4 from the next posterior filament (further one filament posterior). The number of symbionts within cells 1–5, located around the eighth gill filament anterior to the posteriormost bz, did not strictly correspond to their relative spatial locations along the anterior-posterior axis of the gill filaments. In contrast, as indicated by whole-mount FISH (Fig. 1C), host cells in more anterior filaments were predominantly filled with numerous symbionts.

### Morphological characteristics of the apical surface of early colonizing cells

In subsequent SEM observations of the gill filaments across four juvenile individuals, numerous host cells around the eighth filament anterior to the bz were screened. Among the observed cells, three distinct types roughly corresponding to the three stages reconstructed by array tomography were consistently identified, and representative images were shown (Fig. 1I–N).

First, Type 1 cells were surrounded by cells with numerous apical microvilli; however, the Type 1 cells themselves possessed very few microvilli and an isolated and localized protuberance (Fig. 1I). Several bacteria-like structures were visible through the openings in these protuberances (Fig. 1J). Type 2 cells were characterized by a partial protuberance occupying approximately half of the cell surface (Fig. 1K). This structure consisted of a perforated membrane with numerous irregular openings, through which some bacteria-like structures were observed (Fig. 1L). The surrounding area consisted of similar cells and cells with numerous microvilli. Finally, in Type 3 cells, the entire apical surface exhibited a slightly swollen appearance with numerous irregular openings (Fig. 1M). Numerous bacteria-like structures were visible through these openings (Fig. 1N), indicating that the underlying interconnected space maintained continuous contact with the external environment. The surrounding area consisted of similar cells and cells with flattened apical surfaces, a morphology previously reported in mature bacteriocytes in adult mussels [21].

### Genetic variations in symbionts within single early colonizing cells

**Fig. 3:**
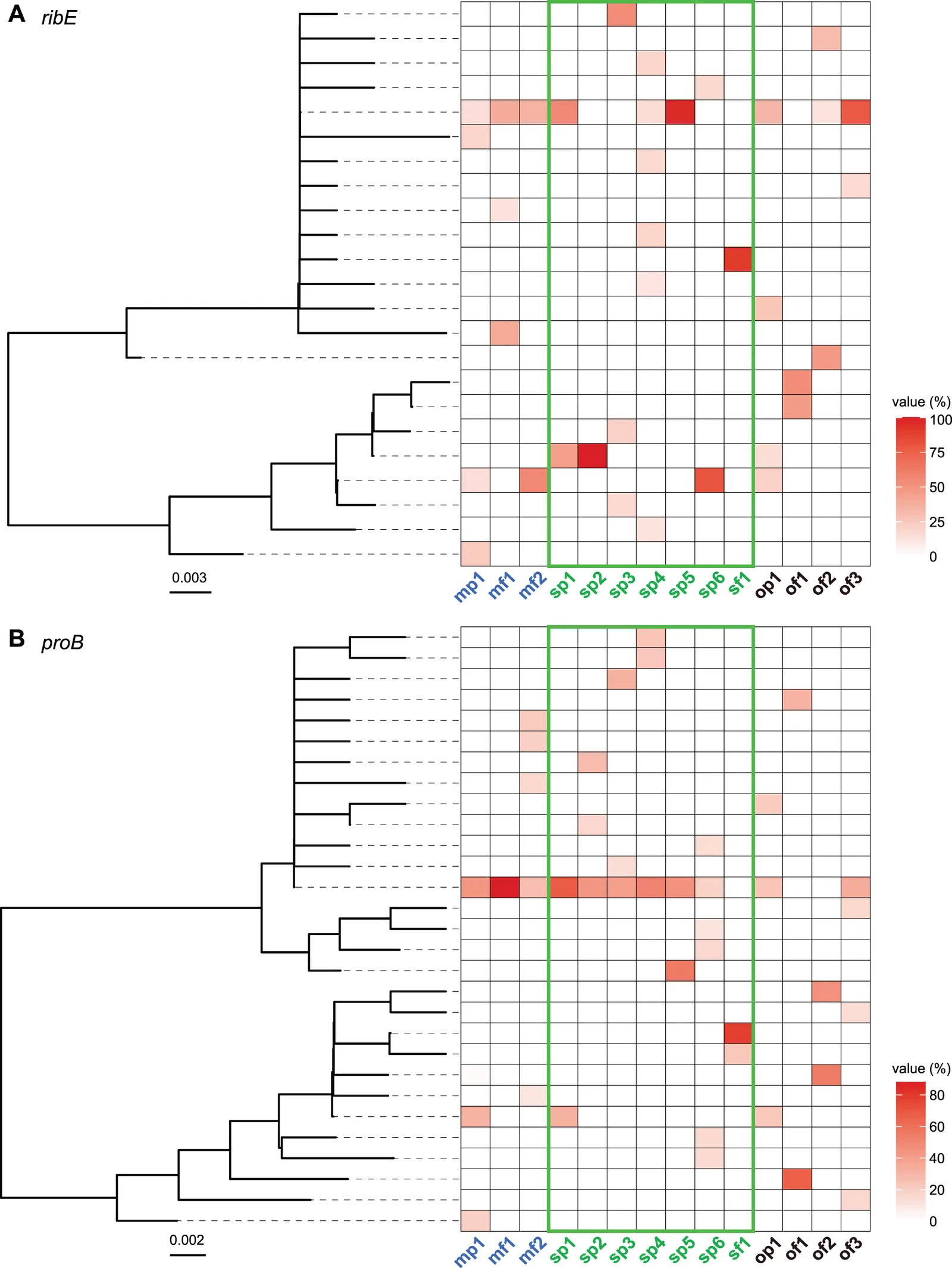
Phylogenetic relationships and relative abundances of symbiont ASVs. (**A, B**) Phylogenetic trees and corresponding heatmaps for amplicon sequence variants (ASVs) of (**A**) *ribE* and (**B**) *proB*. In each panel, the left side displays the molecular phylogenetic tree of detected ASVs. The scale bars below the trees represent nucleotide substitutions per site. The right side shows a heatmap representing the relative abundance (%) of each ASV in the samples. The samples on the horizontal axis are grouped into three categories from left to right: three samples comprising gill regions with multiple (designated as ‘m’) host cells (mp1, mf1, and mf2; blue); seven samples of single (designated as ‘s’) early colonizing cells excised via laser microdissection (sp1–sp6 and sf1; green), indicated with green rectangles; and four reference samples extracted from areas outside (designated as ‘o’) the tissue (op1 and of1–3, black). The second letter of each sample name indicates the embedding method (‘p’ for paraffin and ‘f’ for frozen sections)

To investigate the population structure of symbionts within early colonizing cells, individual cells were recovered using laser microdissection (LMD) from four juvenile individuals, and amplicon sequence variant (ASV) analysis was performed targeting the *ribE* and *proB* genes. The phylogenetic relationships between the ASVs and their relative abundances in each sample are shown as heatmaps (Fig. 3). Overall, the ASV phylogenies for both *ribE* and *proB* were broadly divided into two major clades. Furthermore, although some ASVs were shared across multiple samples for both genes, the detected ASVs generally varied considerably from sample to sample.

Among the three samples comprising gill regions with multiple cells (samples mp1, mf1, and mf2), *ribE* analysis revealed that sample mf1 contained three ASVs belonging to only one of the two major clades, whereas samples mp1 and mf2 contained multiple ASVs (four and two, respectively) spanning both clades (Fig. 3A). Similarly, *proB* analysis showed that sample mf1 was dominated by a single ASV, whereas samples mp1 and mf2 contained multiple ASVs (three and five, respectively) spanning both clades (Fig. 3B). When multiple ASVs were detected, their amplicon read proportions were generally comparable, with no strong bias toward any specific ASV in either *ribE* or *proB*.

In the seven samples in which single cells were excised via LMD (samples sp1–6 and sf1), *ribE* analysis detected only a single ASV in three samples (sp2, sp5, and sf1). The other four samples contained multiple ASVs (two-six) spanning both clades. Among these, one sample (sp6, containing two ASVs) showed a skewed relative abundance toward one ASV, whereas the remaining three showed comparable read proportions (Fig. 3A). For *proB*, multiple ASVs (two-six) were observed in all seven single-cell samples. In five of these samples (sp2–5 and sf1), the ASVs were restricted to only one of the two major clades. Within this restricted group, one sample (sf1) was strongly dominated by one of its two ASVs, whereas the other samples exhibited comparable read proportions. The ASVs in the remaining two samples (sp1 and sp6) spanned both major clades with comparable read proportions (Fig. 3B). The patterns of ASV variation and clade distribution within individual single cells were generally discordant between these two loci.

As a reference, we also extracted DNA from areas outside the tissue regions and performed a similar PCR amplification. Amplification was observed for both *ribE* and *proB*, and multiple ASVs were detected for both genes (Fig. 3). Because no SYBR Green signals were observed in these areas, this amplification likely resulted from cell-free DNA leaked from the tissue during sample preparation, rather than from intact bacterial cells. Although the impact of this leakage on the single-cell samples excised from within the tissue via LMD is expected to be minimal, the possibility of contamination during SYBR Green staining or microscopic observation cannot be completely ruled out. Therefore, to verify the genetic heterogeneity of symbionts within single cells in situ without the risk of extraction-based contamination, we conducted direct-geneFISH analysis.

### Spatial distribution of symbiont subpopulations within individual bacteriocytes

**Fig4.**
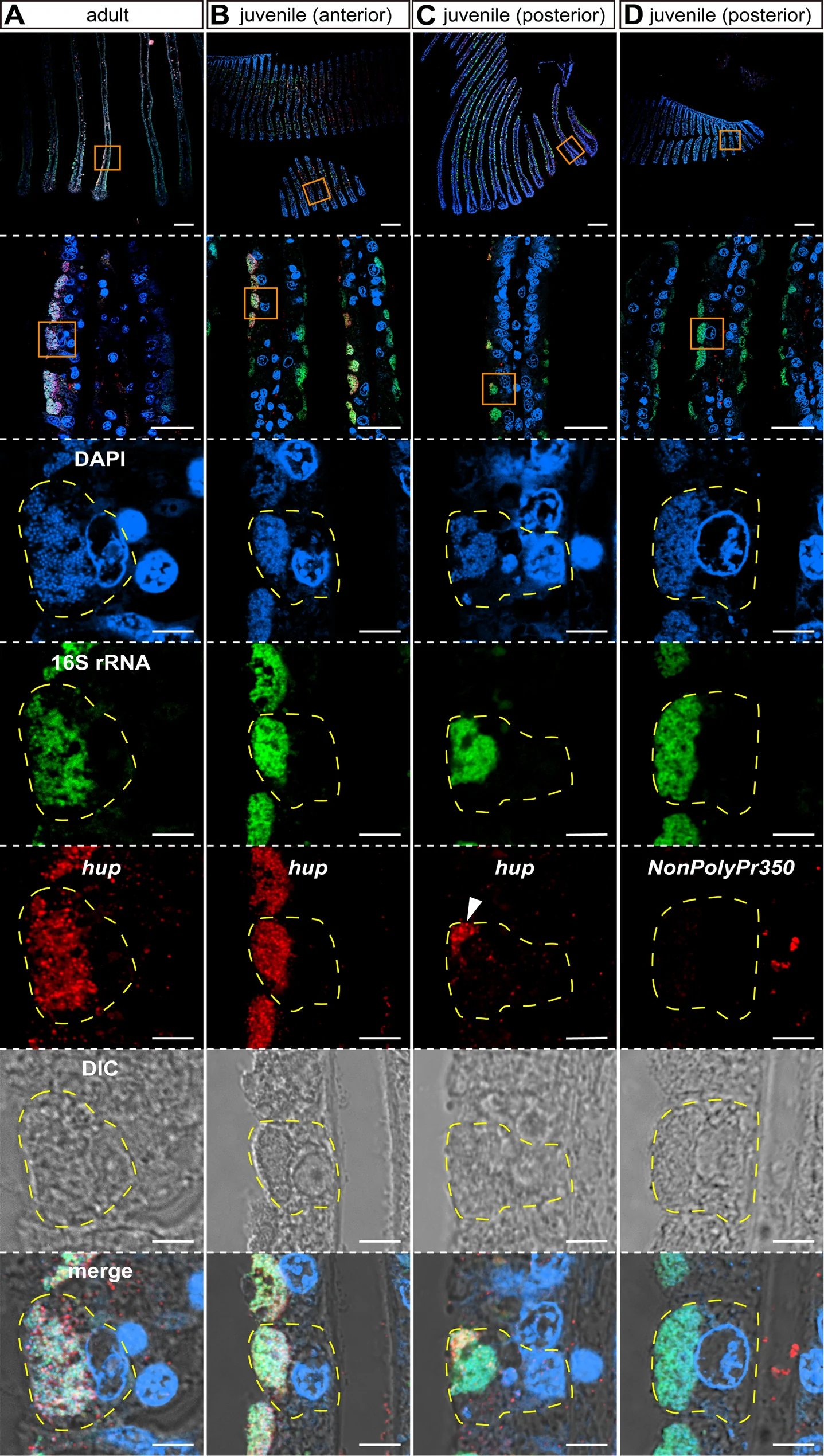
Spatial distribution of symbiont subpopulations within a single host cell. Direct-geneFISH targeting the *hup* gene cluster was performed to visually verify the coexistence of diverse symbiont populations within individual host cells in situ. Fluorescence signals for host and symbiont DNA (DAPI), symbiont 16S rRNA, and the *hup* gene cluster are shown in blue, green, and red, respectively. The figure consists of four columns representing different samples: (**A**) An adult gill, (**B**) The anterior region of a juvenile gill, and (**C, D**) The posterior-most region of a juvenile gill (containing early colonizing cells). Each column displays seven vertically arranged panels; from top to bottom, these represent a low-magnification merged fluorescence image, a higher-magnification view of the region indicated by an orange rectangle in the first panel, and a sequence of five highly magnified panels focusing on a single cell (outlined by a yellow dashed line), showing the DAPI, 16S rRNA, *hup* (or NonPolyPr350), and differential interference contrast (DIC) images, followed by a fully merged image of these four. In the adult (**A**) and juvenile anterior (**B**) gill cells, *hup* signals completely colocalized with the 16S rRNA and DAPI signals within individual *hup*-positive cells. In the early colonizing cells of the juvenile posterior gill (**C**), *hup* signals overlapped with only a portion of the 16S rRNA and DAPI signals (indicated by white arrowheads). (**D**) No specific signals were detected for the NonPolyPr350 probe (red). In all juvenile gill section images (panels B–D), the posterior side is oriented to the right. Scale bars: 100 µm (top row), 25 µm (second row), and 5 µm (third and subsequent rows)

To visually verify the coexistence of diverse symbiont populations within single host cells in situ, direct-geneFISH analysis targeting the *hup* gene cluster was performed, and genomic heterogeneity was examined independently of ASV analysis. In the gill from an adult mussel (Fig. 4A), DAPI stained both the host cell nuclei and the symbionts. The 16S rRNA signals, representing the total symbiont population, completely overlapped with the DAPI signals (excluding the host cell nuclei), and cells exhibiting these signals were identified as bacteriocytes. Consistent with a previous report [8], *hup* signals were detected only in a subset of bacteriocytes, exhibiting a mosaic distribution of the *hup*-harboring subpopulation across the tissue. In the juvenile gills (examined in four individuals), the anterior region (Fig. 4B) exhibited a pattern identical to that of adults: *hup* signals were detected in only a subset of bacteriocytes, and within these cells, the *hup* signals completely colocalized with 16S rRNA and DAPI signals. In contrast, a distinct pattern was observed in the early colonizing cells within the posterior-most region of the juvenile gills (Fig. 4C). Although the overall tissue still contained a mixture of *hup*-positive and -negative cells, within the individual *hup*-positive cells in this region, the *hup* signals overlapped with only a portion of the 16S rRNA and DAPI signals, visually confirming the coexistence of distinct symbiont subpopulations within a single host cell. The negative control using the NonPolyPr350 probe in the posterior region of juvenile gills yielded no specific fluorescence (Fig. 4D); only minor, scattered spots representing non-specific background were observed.

## Discussion

### Cellular remodeling during initial colonization

Here, we provide the first high-resolution 3D ultrastructural characterization of the initial symbiont colonization and bacteriocyte development in *B. septemdierum*. By combining three-dimensional ultrastructural analysis with single-cell amplicon sequencing and direct-geneFISH, we captured the dynamic progression of early symbiotic events that were previously elusive.

As in vivo observations at the electron microscopic level are currently not possible, studying the spatial arrangement of gill filaments in deep-sea mussels, which display a continuum of symbiotic stages from the initial stage to maturity, provides a powerful approach for capturing the temporal progression of the colonization process using fixed samples. Previous two-dimensional ultrastructural studies of closely related host species (e.g., *B. azoricus* and *B. puteoserpentis*) demonstrated that symbiont colonization initiates in the posterior part of the gill (i.e., around the eighth filament anterior to the posteriormost bz) and is accompanied by the loss of host microvilli and cell hypertrophy [12]. The morphological characteristics of cells in the colonization stages observed in our 3D and SEM analyses, namely, the loss of microvilli, as well as cell flattening and enlargement, are consistent with these 2D observations and with their interpretation as representing a chronological progression of symbiont colonization [12]. Furthermore, our quantitative results revealed a descriptive positive correlation between host cell volume and the number of symbionts engulfed within the cavity. Therefore, we conclude that the sequence of structural differences observed in our study represents the actual chronological progression of host cellular remodeling during the initial symbiont colonization. The progression of symbiont counts across cells 1–5 did not strictly correspond to a linear spatial sequence along the gill filaments. This indicates that early colonization takes place across a spatial region with mixed cellular stages around the eighth filament, rather than following a strict linear order along the filaments. In contrast, host cells in more anterior filaments were predominantly filled with numerous symbionts, representing a state close to mature bacteriocytes.

The acquisition of symbiotic bacteria from the environment by animal host cells is generally understood to rely on the co-option of phagocytosis [1, 4]. Indeed, in the closely related deep-sea mussel *G. japonicus*, gill epithelial cells have been shown to acquire symbionts through phagocytosis [17]. However, our 3D data revealed that the cellular morphological changes during the initial colonization of symbiotic bacteria in *B. septemdierum* are accompanied by a highly dynamic and atypical uptake process. This process is distinct from typical “zippering” phagocytosis, in which the cell membrane tightly encloses individual targets into closed vesicles within minutes [2, 36], and is also fundamentally different from macropinocytosis, which involves the rapid bulk engulfment of extracellular fluid and particles to form closed intracellular vacuoles [37]. Instead, the gill epithelial cells of *B. septemdierum* initially form a relatively large, spacious cavity beneath the apical surface that accommodates only a few bacteria and remains open to the external environment. As the host cell slowly hypertrophies over time, this open cavity gradually expands laterally along the apical surface rather than deepening toward the basal side, while the bacterial population within it increases to dozens of cells. During this process, the expanded cavity undergoes compartmentalization. Although 2D cross-sections of more advanced colonization stages (e.g., cells 4 and 5) appear to show multiple isolated cavities, our 3D reconstruction demonstrated that they constitute a single interconnected cavity consisting of several compartments. As this compartmentalization progresses further, smaller chambers enclosing only a single symbiont are formed. This protracted sequence of cellular remodeling contrasts with acute microbial engulfment and is reminiscent of the gradual epithelial morphogenesis associated with symbiont colonization observed during the establishment of the squid-*Vibrio* symbiosis [5]. More broadly, recent studies in diverse animal symbioses have emphasized that specialized symbiotic environments do not pre-exist fully formed, but are progressively constructed through dynamic, host-driven cellular and tissue remodeling (e.g. [10, 38]). Although the scale and biological systems differ—ranging from multicellular organogenesis in insects [10, 38] to single-cell membrane architecture in deep-sea bivalves—our findings illustrate a parallel cellular principle wherein the host actively creates a specialized, compartmentalized niche to accommodate and manage its microbial partners. Moreover, a particularly unique feature of this complex remodeling is the formation of a dome-like “roof” over the cavity, consisting of a perforated membrane with numerous openings. Through these mesh-like openings in the apical roof, the internal compartments and chambers maintain continuous contact with the external environment. This sequential cellular remodeling will help elucidate the developmental origin of the mature bacteriocyte architecture, demonstrating how its intricate, externally open chamber network [21] is constructed from a single initial cavity. However, the exact formation process of the highly complex passages composed of overlapping membranes (Fig. S1), occasionally observed in adult bacteriocytes [21], remains unclear.

### Symbiont population dynamics and intracellular purification

Our population-level analyses revealed that symbiont populations exhibit genetic heterogeneity during the initial stages of colonization. ASV analysis of the *ribE* and *proB* genes from single cells isolated via laser microdissection (LMD) indicated that cells in the early colonization stages contained multiple ASVs (ranging from two to six per cell). Phylogenetic analyses showed that these ASVs generally fell into two major clades, with multiple ASVs spanning both clades frequently co-detected within single cells. The coexistence of such highly divergent sequences is unlikely to be explained solely by PCR or sequencing artifacts, which typically generate only closely related minor variants. Furthermore, the discordance in ASV patterns between the *ribE* and *proB* loci within individual single cells implies that the early intracellular population is not entirely clonal, but rather consists of genetically diverse strains. These findings collectively suggest the presence of phylogenetically diverse symbiont strains that co-colonize individual host cells. However, because multiple ASVs were also detected in the reference samples outside of the tissue regions, the possibility of contamination from cell-free DNA leakage during sample preparation could not be completely excluded. To provide independent in situ support and overcome this extraction-based limitation, we evaluated a distinct genetic metric: the presence or absence of the *hup* genomic locus, visualized via direct-geneFISH. In host cells in the early stages of symbiont colonization, we found that the *hup* signals overlapped with only a portion of the total symbiont population, as visualized by the 16S rRNA and DAPI signals. This partial overlap confirmed the coexistence of *hup*-positive and *hup*-negative symbiont subpopulations within individual host cells. Amplicon sequencing of microdissected samples provides high-resolution sequence polymorphism data but remains potentially vulnerable to contamination from DNA leakage. Conversely, direct-geneFISH provides robust, contamination-free in situ validation but is limited to detecting genomic locus presence/absence rather than specific nucleotide variants. By combining these two complementary approaches, we address their respective methodological limitations and provide mutually supportive evidence that multiple genetically distinct symbiont strains coexist within a single early-colonizing host cell. Thus, this combined evidence of initial genetic heterogeneity challenges the hypothesis that host cells acquire only a single bacterial cell from the environment at the onset of symbiont colonization [21]. Instead, our data suggest that colonization may be initiated by the concurrent acquisition of multiple symbiont strains. Given that each mature bacteriocyte harbors a nearly clonal population within interconnected chambers [21], this initial heterogeneous mixture may undergo a process of intracellular selection or purification at some stage during host cell maturation to establish a final clonal population.

We can then speculate how this purification process corresponds to the observed cellular remodeling. Owing to the optical resolution limits of FISH, it is challenging to definitively link the fluorescent signals to precise subcellular structures. Consequently, it remains unclear whether these distinct bacterial subpopulations are segregated into specific large compartments, enclosed within smaller chambers, or distributed independently of these physical boundaries. However, our FISH observations revealed that the *hup*-positive symbionts were not randomly scattered but tended to localize within specific regions of the host cell. This uneven distribution may reflect the ongoing processes of cavity compartmentalization or small-chamber formation, suggesting that some form of spatial sorting may already be operating at these relatively early stages. In other horizontally transmitted animal–microbe associations, the sorting and purification of specific symbiont strains from environmental mixtures typically occur within distinct extracellular spaces, such as the epithelial crypts of the squid *Euprymna scolopes* [11] or the specialized constricted region in the midgut of the stinkbug *Riptortus pedestris* [10]. In contrast, our findings suggest that *B. septemdierum* executes this sorting and purification process within a unique intermediate environment, a pseudo-intracellular space that maintains connections to the exterior while undergoing dynamic spatial partitioning by individual host cells.

Recent metagenomic analyses have demonstrated that symbiont populations within individual mussels of the closely related species *B. brooksi* become highly clonally purified as the host grows [9]. The authors proposed that this purification is driven by serial founder effects during continuous “self-infection” of newly formed gill tissues. Our 3D morphological data reveal an intracellular phenomenon that may contribute to such a purification process. The initial cavity, containing multiple symbiont strains, progressively partitions into separate compartments and narrower chambers as the host cell matures. Although these structures maintain connections with the external environment through apical openings, preventing complete physical isolation and potentially allowing the passage of bacteria, this intricate spatial partitioning could still restrict the free movement and continuous bulk mixing of the bacterial population. We propose that this physical subdivision, supported by the localized FISH signals, may promote spatial isolation at the microscale level. Within these confined microenvironments, bacteria may undergo local competition and selection, ultimately driving the intracellular purification into a single clone. This morphologically restricted cellular environment may act as a physical “bottleneck” during each self-infection cycle, facilitating the serial founder effects proposed in a previous study on *B. brooksi* [9], although further population genomic studies are required to confirm whether this macro-level dynamic also occurs in *B. septemdierum*.

### Refinement of the symbiont colonization model

To explicitly clarify how our present findings refine the previously proposed model for *B. septemdierum* [21], we compare the core elements of both models. The fundamental structural framework established in the previous model—specifically, that host membranes undergo progressive reorganization to construct an open-ended chamber network maintaining continuous connectivity with the external environment, and that mature bacteriocytes host largely uniform symbiont populations—is consistent with our 3D ultrastructural reconstructions of early-stage host cells and direct-geneFISH observations in mature host cells. However, our multimodal analyses substantially update the early events and population dynamics during colonization. While the previous hypothesis assumed a simple sequence starting from the uptake of a single founding bacterial cell followed by continuous membrane branching [21], our 3D reconstructions reveal a much more specific and protracted cellular remodeling process. Early colonization proceeds through distinct, specialized morphological stages: the formation of a localized apical protuberance and underlying cavity, its lateral expansion accompanied by a perforated, mesh-like apical roof, progressive compartmentalization of the cavity, and the formation of narrower chambers via constriction. Concurrently with this dynamic specialized morphogenetic sequence, host cells accommodate a genetically heterogeneous mixture of symbiont strains rather than a single bacterium. Thus, intracellular clonal purification is unlikely to be established at the outset by a single-cell infection bottleneck; instead, we propose that it may be progressively facilitated by spatial partitioning during this unique cellular remodeling process.

## Conclusions

In this study, we addressed the fundamental question of how the unique symbiotic state of *Bathymodiolus septemdierum*—characterized by an intricate, externally open cellular architecture coupled with an intracellularly uniform symbiont population—is established during early colonization. As discussed above, our integrated 3D ultrastructural and single-cell molecular analyses demonstrate that initial colonization is driven by a dynamic, multi-stage cellular remodeling process accompanied by the concurrent acquisition of a genetically heterogeneous symbiont population, thereby revising the previously proposed single-cell uptake model. These findings point to a close association between host cellular morphodynamics and symbiont population structuring, suggesting that intracellular clonal purification progresses alongside host spatial partitioning during bacteriocyte maturation. While the exact mechanisms driving this intracellular sorting process remain a compelling subject for future study, our integrated approach provides a critical framework for deciphering how animal hosts dynamically reorganize their cells and manage microbial populations to establish stable endosymbioses. Given the wide diversity of symbiotic modes within the subfamily Bathymodiolinae [13–15], the specific cellular remodeling and symbiont population dynamics observed in *B. septemdierum* may not be applicable to all bathymodioline mussels. However, a detailed and accurate understanding of this diversity is crucial for elucidating the universal mechanisms by which stable symbiotic relationships are established. Integrating high-resolution cellular and structural analyses with single-cell molecular profiling, as demonstrated in this study, can provide essential knowledge toward this goal, highlighting the underlying relationship between host cellular remodeling and symbiont population dynamics.

## Electronic supplementary material

Below is the link to the electronic supplementary material.

Supplementary Material 1: Figure S1. Ultrastructure and schematic representation of a mature bacteriocyte in adult *Bathymodiolus septemdierum*

Supplementary Material 2: Figure S2. Confirmation that nucleic acid signals in bacteriocytes exclusively represent the symbionts

Supplementary Material 3: Table S1. PCR primer sequences for direct-geneFISH probe synthesis

Supplementary Material 4: Movie S1. Tomographic three-dimensional reconstruction of the bacteriocyte ultrastructure from thin sections of cell 1. Movie S2. Tomographic three-dimensional reconstruction of the bacteriocyte ultrastructure from thin sections of cell 2. Movie S3. Tomographic three-dimensional reconstruction of the bacteriocyte ultrastructure from thin sections of cell 3. Movie S4. Tomographic three-dimensional reconstruction of the bacteriocyte ultrastructure from thin sections of cell 4. Movie S5. Tomographic three-dimensional reconstruction of the bacteriocyte ultrastructure from thin sections of cell 5

## Data Availability

The datasets supporting the conclusions of this article are included within the article and its additional files. Further information and requests for data should be directed to and will be fulfilled by the corresponding author.
