# Supplementary material for: Dynamic cellular remodeling and symbiont genetic heterogeneity during early bacterial colonization in the deep-sea mussel Bathymodiolus septemdierum
Source: Zoological Lett. 2026 Aug 21;12:18. doi: 10.1186/s40851-026-00271-w (PMC13651418; doi:10.1186/s40851-026-00271-w)
Supplement: Supplementary file 1 [file 40851_2026_271_MOESM1_ESM.pdf]

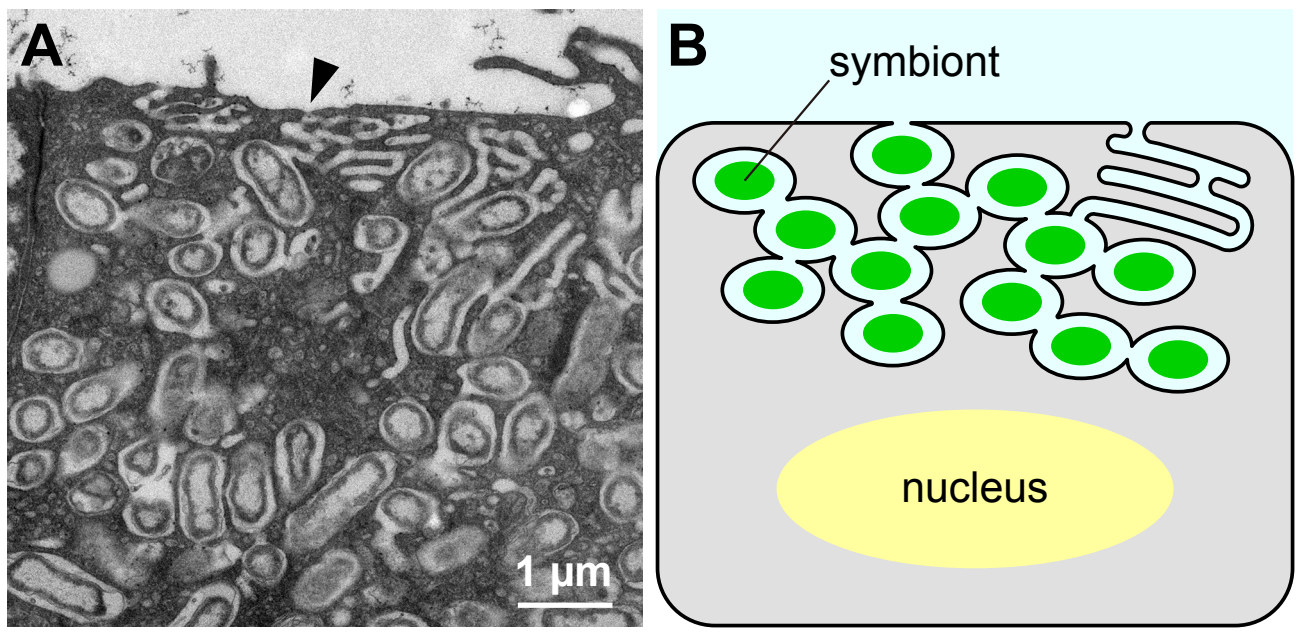

Figure S1. Ultrastructure and schematic representation of a mature bacteriocyte in adult *Bathymodiolus septemdierum*. (A) Transmission electron micrograph (TEM) of a representative mature bacteriocyte in an adult mussel (apical side up). Black arrowhead indicates an opening to the external extracellular space via occasionally observed multi-layered membranous passages. **(B)** Corresponding schematic diagram illustrating the interconnected chamber network and its pathways connecting to the external environment.
