# Supplementary material for: Dynamic cellular remodeling and symbiont genetic heterogeneity during early bacterial colonization in the deep-sea mussel Bathymodiolus septemdierum
Source: Zoological Lett. 2026 Aug 21;12:18. doi: 10.1186/s40851-026-00271-w (PMC13651418; doi:10.1186/s40851-026-00271-w)
Supplement: Supplementary file 2 [file 40851_2026_271_MOESM2_ESM.pdf]

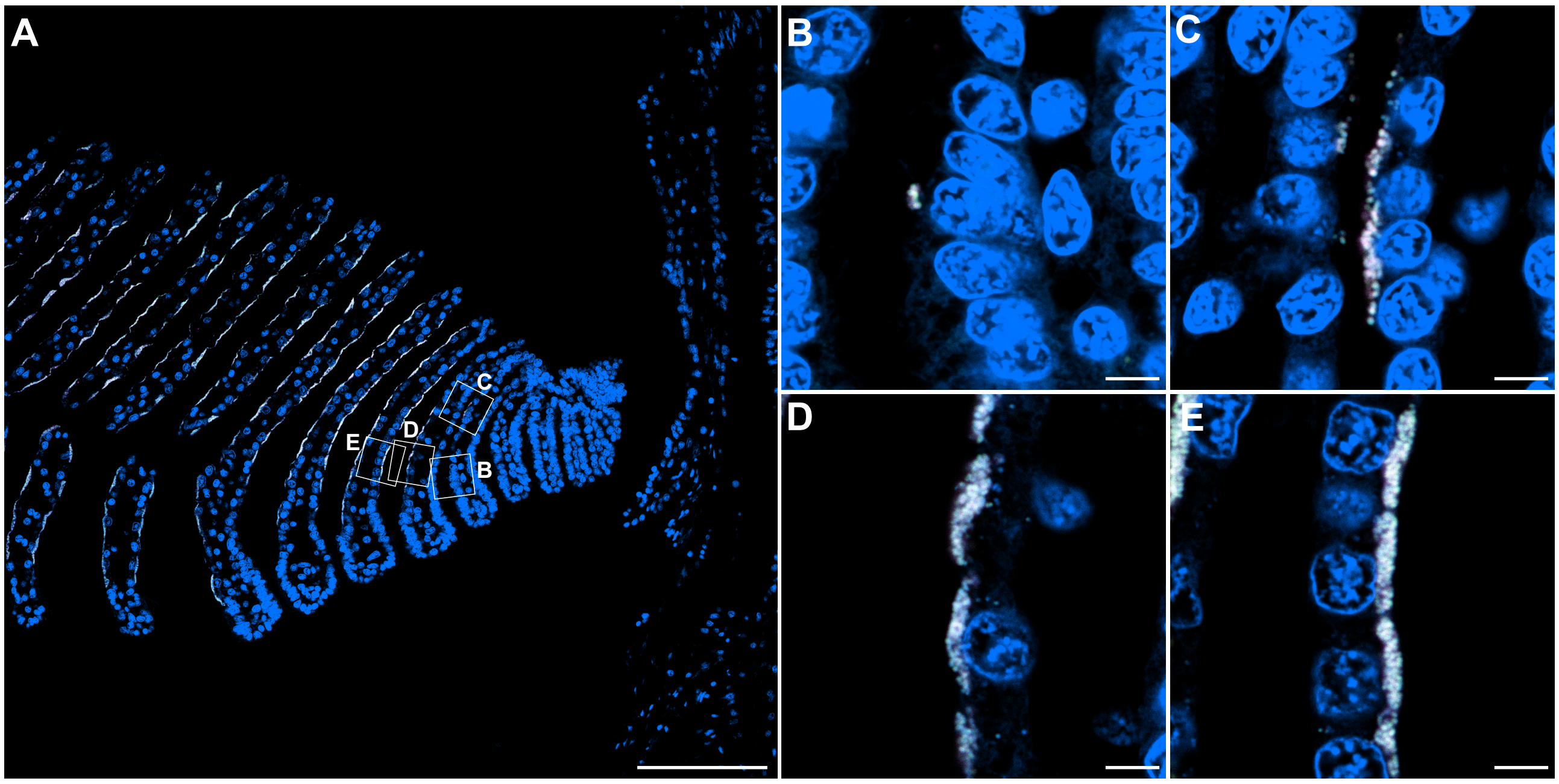

Figure S2. Confirmation that nucleic acid signals in bacteriocytes exclusively represent the symbionts. (A) Low-magnification overview of the posterior region of the juvenile gill. White rectangles indicate the regions magnified in (B–E). **(B-E)** High-magnification images of bacteriocytes. In all panels, the fluorescence signals of DAPI (blue), the symbiont-specific 16S rRNA probe (green), and the general bacterial probe EUB338 (magenta) are merged. The overlap of the three signals appears white. Within the bacteriocytes, no spots positive solely for DAPI and EUB338 were observed. This visually demonstrates that all bacteria present are symbionts, confirming the absence of non-symbiotic bacteria. In all panels, the posterior side is oriented to the right. Scale bars: 100 μm (A) and 10 μm (B–E). This validates the use of nucleic acid staining as a reliable marker for the specific isolation of symbionts by laser microdissection.
